# Influence of a Siloxane-Modified DOPO Derivative on the Properties of Polyurethane Cationomer Coatings

**DOI:** 10.3390/ma18040789

**Published:** 2025-02-11

**Authors:** Łukasz Byczyński, Mariusz Szołyga, Piotr Król

**Affiliations:** 1 Faculty of Chemistry, Rzeszow University of Technology, Al. Powstańców Warszawy 6, 35-959 Rzeszow, Poland; pkrol@prz.edu.pl; 2 Poznan Science and Technology Park, Adam Mickiewicz University Foundation, Rubież 46, 61-612 Poznan, Poland; mariusz.szolyga@ppnt.poznan.pl

**Keywords:** polyurethane, phosphorus, waterborne, cationomer, flame retardancy, DOPO

## Abstract

Waterborne polyurethane cationomer coatings modified with 1,3-bis(3(3-(propoxy-2-ol-)-9,10-dihydro-9-oxa-10-phosphaphenanthrene-10-oxide)-3-propyloxy))tetramethyldisiloxane (TMDS–AGE–DOPA) containing phosphorus and silicon atoms were obtained. Their structures were confirmed by Fourier transform infrared (FTIR) spectroscopy. The effect of TMDS–AGE–DOPA on thermal properties, flame retardancy, and surface characteristics (gloss, contact angle, surface free energy), as well as performance properties (hardness, impact resistance), was investigated. A coupled TG-FTIR technique was employed for evolved gas analysis. Thermal stability decreased with the addition of the modifier, while the glass transition temperature increased from −19 to 25 °C. The modifier improved the flame retardancy of the material by shifting the peak temperature of the heat release rate (T_PHRR_) to lower values. The gloss of the coatings was very high (>90 GU at all angles studied), although it decreased with increasing modifier content. The presence of phosphorus moieties from the modifier enhanced hydrophilicity, raising surface free energy (SFE) from 37.9 to 44.0 mJ/m^2^. The coatings are soft materials with a Persoz hardness in the range of 0.05–0.32. The modifier increased hardness but reduced impact strength. The obtained cationomers can be applied as environmentally friendly coatings on hydrophilic surfaces such as textiles, glass, or wood.

## 1. Introduction

Research into environmentally friendly materials, such as low-VOC (volatile organic compound) coatings, is essential to reduce environmental impact and improve human health. Polyurethane waterborne coatings are popular for their excellent mechanical properties, versatility, and reduced toxicity compared to solvent-based alternatives [1]. However, polyurethanes (PU) are flammable, which poses a safety risk in certain applications. Modifying coatings with flame retardants can improve their fire resistance while preserving their eco-friendly advantages.

The use of halogen-free flame retardants is limited due to the release of toxic and corrosive gases during degradation [2,3]. Therefore, research is being carried out on the modification of polymers with compounds that do not contain halogen atoms. The most widely used halogen-free flame retardants include those containing phosphorus, nitrogen, boron, and silicon atoms [4,5]. Currently, halogen-free flame retardants are widely used to enhance the fire resistance of waterborne polyurethane and can be classified into two main categories: addition-type and reactive-type flame retardants. Reactive flame retardants are integrated into polymer chains during polymerization, ensuring permanent flame resistance without altering the physical properties of the material or causing migration to the surface. These retardants, chemically bound to polymers, offer higher thermal stability than additive flame retardants [6,7].

Among halogen-free flame retardants, phosphorus-based flame retardants are particularly effective because of their high efficiency and low toxicity. They work in both the condensed and gas phases. In the condensed phase, phosphorus-based flame retardants promote char formation, creating a protective insulating layer that reduces heat and oxygen access. During combustion, they form stable poly(metaphosphoric acid), which condenses into pyrophosphate structures (P-O-P) and releases water (H_2_O), cooling the surface. In the gas phase, they act as free radical scavengers, disrupting flame-sustaining reactions and diluting the oxidizing gas phase, thereby suppressing fire spread. These mechanisms collectively limit the decomposition of materials and reduce the amount of heat released. In polyurethanes, phosphorus accelerates initial degradation at low temperatures due to the weaker P-O bond (149 kJ/mol) compared to the C-O bond (256 kJ/mol). However, this degradation stabilizes in later stages as char formation inhibits further combustion [8,9].

Among the phosphorus-containing flame retardants used to modify waterborne polyurethanes, 9,10-dihydro-9-oxa-10-phosphaphenanthrene-10-oxide (DOPO) and its derivatives have received considerable attention due to their high flame retardancy efficiency, high reactivity, and low loading levels. These compounds exhibit dual activity in both the gas and condensed phases, demonstrating a high char-forming ability. However, while DOPO enhances fire safety, its rigid heterocyclic structure can increase tensile strength but reduce the flexibility of polyurethanes [10,11,12,13,14,15]. Wang et al. [16] used 9,10-dihydro-9-oxa-10-[N,N-bis-(2-hydroxyethylamino-methyl)]-10-phosphaphenanthrene-10-oxide (DOPO-DAM) to modify polyurethane anionomers. The addition of the modifier increased the flame resistance. However, it was noticed that the content of the modifier, more than 3%, caused a decrease in the tensile strength and Young’s modulus. This is due to the presence of rigid DOPO structures, which hinder the crystallization of the hard segment and restrict the interreaction of polyurethane chains. Zhou et al. [17] studied waterborne poly(urethane-siloxane) anionomers modified with DOPO-DAM and concluded that the introduction of modifier significantly improved the flame retardancy of coated polyester fabric. Additionally, the introduction of siloxane increased the hydrophobicity of the obtained materials. Cui et al. [18] investigated polyurethane anionomers modified by PHID (4-DOPO-((3-hydroxypropyl)imino)methyl)phenol, and hydroxyl silicone oil (HO-Si) as a silicon-based additive. These modifications significantly improved flame retardancy, physical properties, and thermal stability.

Polyurethane cationomers, due to their properties, can find wide applications in industry, including as adhesives and protective coatings for glass, textiles, and wood. Hence, studies on such materials hold great scientific and practical significance. However, research on waterborne polyurethanes modified with flame retardants mainly concerns polyurethane anionomers, while the modification of cationomers has so far been rarely reported in the literature. Our previous studies have shown that the use of a DOPO siloxane derivative to modify UV-cured waterborne poly(urethane-acrylic) cationomers had a beneficial effect in reducing the flammability and hydrophilicity of the cured coating [19].

In the presented work, we considered it purposeful to check the flammability and thermal properties of the coatings obtained from linear polyurethane cationomers with the participation of a new polyol component containing phosphorus atoms and siloxane groups by introducing a DOPO derivative of 1,3-bis(3(3-(propoxy-2-ol-)-9,10-dihydro-9-oxa-10-phosphaphenanthrene-10-oxide)-3-propyloxy))tetramethyldisiloxane (TMDS–AGE–DOPA). With the potential application of the cationomers as environmentally friendly protective coatings in mind, we considered it advisable to investigate also their selected surface and performance properties.

## 2. Materials and Methods

Isophorone diisocyanate 98% (IPDI, Acros Organics, Geel, Belgum), N-methyldiethanolamine (NMDA, Acros Organics, Geel, Belgum), 1,4-butanediol (BD, Sigma-Aldrich, Darmstadt, Germany), formic acid 99% (HCOOH, Chempur, Piekary Śląskie, Poland), and dibutyltin dilaurate (DBTDL, Sigma-Aldrich) were used without further purification. Poly(oxytetramethylene)diol (PTMO, Mn = 1000) was purchased from Merck and dried in a vacuum oven at 105 °C for 2 h before use. 1,1,3,3-tetramethyldisiloxane (TMDS), Karstedt catalyst (2% in xylene), and allyl-glycidyl ether (AGE) were purchased from Aldrich (Darmstadt, Germany). DOPO (9,10-dihydro-9-oxa-10-phosphaphenanthrene-10-oxide) was purchased from TCI Chemicals (Tokyo, Japan), while H_2_O_2_ (30% in H_2_O), THF, and acetone were purchased from Chempur. All reagents were used without further purification. DOPA (10-hydroxy-9,10-dihydro-9-oxa-10-phosphaphenanthrene-10-oxide) was synthesized according to the procedure described elsewhere [20].

### 2.1. Synthesis of TMDS–AGE

**Figure 1:**
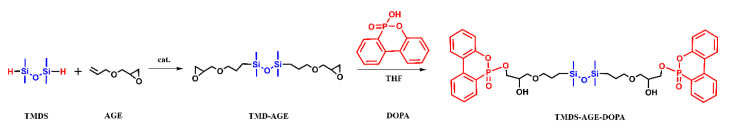
Synthesis scheme of TMDS–AGE and TMDS–AGE–DOPA.

The synthesis of 1,3-bis(3-glycidyloxypropyl)tetramethyldisiloxane (TMDS–AGE) was carried out according to the following procedure. In a 250 mL three-neck flask equipped with a magnetic stir bar, thermometer, and reflux condenser, 34.6 g (0.257 mol) of 1,1,3,3-tetramethyldisiloxane (TMDS) and 67.62 g (0.592 mol; 15% excess) of allyl-glycidyl ether (AGE) were placed. The solution was stirred, and 70 µL of Karstedt’s catalyst was added. After a while, the temperature of the system slowly increased and finally reached 140 °C (after reaching 90 °C, an ice bath was placed under the flask). When the temperature began to drop, the ice bath was removed and the solution was allowed to cool to room temperature overnight. The next morning, FTIR analysis showed complete conversion of Si-H bonds, which confirms the complete conversion of the organosilicon substrate to the glycidyl derivative. Next, the solution was filtered through activated carbon and silica gel to remove catalyst residues. The filter layer was washed with petroleum ether to maximize process efficiency. The collected filtrate was then transferred to a single-necked flask and placed on a rotary evaporator, thereby removing petroleum ether and excess AGE. Evaporation of the volatile ingredients gave a pure product as a colorless oil characterized by low viscosity (yield 98%, 91.2 g). The chemical structure of TMDS–AGE is presented in Figure 1. The formation of the desired product was verified by NMR and FTIR analysis:

**^1^H NMR** (CDCl_3_; 300 MHz,) δ: 0.00 and 0.02 (Si-CH_3_, 12H), 0.46 (Si-CH_2_, 4H), 1.54 (CH_2_-CH_2_, 4H), 2.55 and 2.73 (epoxy-CH_2_, 4H), 3.08 (epoxy-CH, 2H), 3.33 (CH_2_-CH_2_-O, 4H), 3.64 (CH-CH_2_-O, 4H) ppm.

**^13^C NMR** (CDCl_3_; 75 MHz) δ: 0.00 (Si-CH_3_), 13.95 (Si-CH_2_), 23.30 (CH_2_-CH_2_), 43.94 (epoxy-CH_2_), 50.61 (epoxy-CH), 71.15 (CH_2_-CH_2_-O), 74.02 (CH-CH_2_-O) ppm.

**^29^Si NMR** (CDCl_3_; 79 MHz) δ: 7.64

**FT-IR** (cm^−1^): 2951 (C–H); 2950-2800 (-CH_2_) 1257 and 826 (Si-CH_3_); 1104 (C-O-C); 1043 (Si-O-Si); 910 (epoxy).

### 2.2. Synthesis of TMDS–AGE–DOPA

In a 250 mL three-neck flask equipped with a magnetic stir bar, thermometer and reflux condenser, 15.618 g (43 mmol) of 1,3-bis(3-glycidyloxypropyl)tetramethyldisiloxane (TMDS–AGE), 20 g (86 mmol) of 10-hydroxy-9,10-dihydro-9-oxa-10-phosphaphenanthrene-10-oxide (DOPA), and 150 mL of THF were placed. The solution was stirred and heated to 60 °C. Over time, the DOPA precipitate begins to disappear, and a fully clear solution is obtained—which clearly indicates that DOPA is converted to the product because the DOPA precipitate does not dissolve in THF even after many hours of stirring at elevated temperature. Additionally, the progress of the reaction was analyzed by the FT-IR technique by observing the appearance of a band attributed to the hydroxyl bonds (around 3300 cm^−1^) until a constant value was obtained that did not change over time. The solution was kept at the set temperature and stirred for approximately 48 h (although after 24 h, only symbolic changes in the structure of the FTIR spectrum were observed). Then, to remove the reaction solvent (THF), the solution was poured into a single-necked flask, which was placed on a rotary evaporator. Evaporation gave a pure product as a light yellow, viscous oil with nearly quantitative efficiency (yield 99%, 35.265 g). The chemical structure of TMDS–AGE–DOPA is presented in Figure 1. The obtaining of the compound with the desired chemical structure was determined by FT-IR and NMR spectroscopy:

**^1^H NMR** (CDCl_3_; 300 MHz,) δ: 0.00 (Si-CH_3_, 12H), 0.41 (Si-CH_2_, 2H), 1.48 (CH_2_-CH_2_, 4H), 3.25–3.47 (CH_2_OH, CHOH, CH_2_O, 6H); 3.92 (HCOH, 2H); 4.13 (CH_2_OH, 4H); 7.16–7.92 (C–H_ar_, 16H) ppm.

**^13^C NMR** (CDCl_3_; 75 MHz) δ: 0.00 (Si-CH_3_), 13.75 (Si-CH_2_), 23.01 (CH_2_-CH_2_), 66.28–73.80 (CH_2_O, CHOH and CH_2_OH), 119.74–136.54 and 149.23 (C_ar_) ppm.

**^29^Si NMR** (CDCl_3_; 79 MHz) δ: 7.64

**^31^P NMR** (CDCl_3_;162 MHz) δ: 11.05 (P–O–C^I^); 10.15 (P–O–C^II^).

**FT-IR** (cm^−1^): 3380 (OH); 3061, 1595 and 1480–1430 (C=C-H); 2951 (C–H); 2950–2800 (-CH_2_) 1257 and 837 (Si-CH_3_); 1030 (Si-O-Si); 922 (P=O).

### 2.3. Synthesis of Polyurethane Cationomers Modified with TMDS–AGE–DOPA

**Figure 2:**
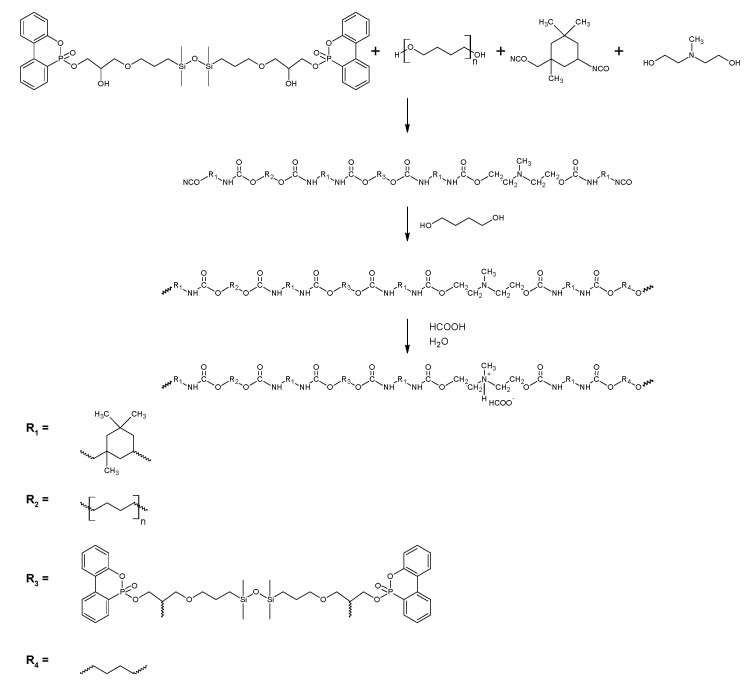
Synthesis scheme of polyurethane cationomer modified with TMDS–AGE–DOPA.

Modified polyurethane cationomers were synthesized in four steps by the acetone process. In the first step, PTMG, TMDS–AGE–DOPA, and NMDA were placed in a 250 mL three-neck flask equipped with a heating mantle, mechanical stirrer, thermometer, reflux condenser, and nitrogen inlet and dissolved in acetone. IPDI was added dropwise to the mixture, and DBTDL was added. The synthesis was carried out at 50 °C, and its progress was monitored using the standard dibutylamine backtitration method. In the second step, BD was added dropwise to the flask, and the synthesis was continued until the complete disappearance of the NCO groups. FTIR spectroscopy was used to monitor this step until the vibration bands around 2257 cm^−1^ were completely absent. In the third step, the polymer was neutralized with HCOOH, which was added in an equimolar amount to NMDA. After 30 min, the stirrer speed was increased to 2000 rpm and water was added dropwise. Dispersing was carried out for 30 min. After this time, the acetone was evaporated in a vacuum evaporator to obtain dispersions containing approx. 30% dry matter. In the same way, an unmodified sample (CP0) was synthesized. In the modified samples, part of PTMO (0.15–0.50 mol) in elastic segments was replaced with TMDS–AGE–DOPA. The synthesis scheme is presented in Figure 2, and Table 1 shows the detailed composition of the individual syntheses. Cationomer dispersions were poured with an applicator on clean substrates and dried first at room temperature for 24 h and then at 65 °C for 72 h. The obtained products were characterized by FTIR spectroscopy and submitted to the tests.

Nuclear magnetic resonance (NMR) spectra were recorded on the Bruker Avance 500^II^ FT NMR spectrometer. The samples were dissolved in CDCl_3_, and a solution with a concentration of approx. 0.2 g dm^−3^ was prepared. Tetramethylsilane (TMS) was used as a standard.

Fourier transform infrared (FTIR) spectra were obtained using a Nicolet iS10 FTIR spectrometer (Thermo Scientific, Waltham, MA, USA) equipped with a diamond ATR accessory. For all measurements, 16 scans were collected at a resolution of 4 cm⁻^1^, covering the spectral range from 4000 to 650 cm⁻^1^.

Differential scanning calorimetry (DSC) measurements were conducted using a DSC1 calorimeter (Mettler-Toledo, Greifensee, Switzerland). Approximately 20 mg of each sample was heated in sealed aluminum crucibles over a temperature range of −100 to 150 °C at a heating rate of 10 °C/min under a nitrogen flow of 50 mL/min. The glass transition temperature (Tg) was determined as the inflection point on the curves obtained during the second heating cycle.

Thermogravimetric analysis (TG) was performed using a Mettler-Toledo TGA/DSC1 analyzer. TG experiments were carried out in nitrogen in the temperature range of 25 to 600 °C with a heating rate of 10 °C min^−1^. Sample weight ca. 15 mg, 50 mL min^−1^ gas flow, and 150 μL open alumina pan were used.

TG-FTIR analyses were performed in nitrogen at 10 °C min^−1^ with the use of the Mettler Toledo TGA/DSC1 instrument, which was online coupled with the Nicolet iZ10 FTIR apparatus (Thermo Scientific) by a transfer line heated at 220 °C. The FTIR spectra of the evolved gases were acquired in the range of 400–4000 cm^−1^.

The flammability of prepared PU materials was assessed using a pyrolysis-combustion flow calorimetry (PCFC) technique. All analyses of PU cationomers were performed on Fire Testing Technology Ltd. (East Grinstead, United Kingdom), FTT0001 MICROCAL. The heating rate (β) was 1.25 °C⋅s^−1^, the pyrolysis temperature range was 75–750 °C, and the combustion temperature was 900 °C. The gas flow was a mixture of O_2_/N_2_ 20/80 cm^3^⋅min^−1^, and the sample weight was 4–5 ± 0.01 mg. The heat release temperature (T_max_), heat release capacity (η_c_), maximum specific heat release rate (HRR_max_), and peak of heat release rate (pHRR) were derived from calorimetric measurements. The tests were repeated three times, and the experimental error in HRR (heat release rate) was ±2%, while the instrumental error in T was 1 °C, and t was 1 s.

Contact angles (CA) were measured with an OCA15 EC optical goniometer (Data Physics, Riverside, CA, USA) with a digital camera installed in the axial extension of the lens. The sessile drop method was employed for the research. Standard liquid drops of water, formamide, and diiodomethane with a constant volume of 1 mL were applied on the surfaces of the samples using a Hamilton microsyringe. Measurements were performed at a temperature of 21 ± 2 °C. The CA values were determined by geometric analysis of the photos taken of the liquid droplets. The result of the contact angle was the mean of five measurements, and the standard deviation was ±2 degrees.

Two distinct methods were utilized to determine the values of surface free energy (SFE). The components of SFE for solids (γ_S_) were calculated using the van Oss–Good (vOG) [21] and Owens–Wendt (OW) [22] models.

According to the van Oss–Good model (Equation (1)), the surface free energy (*γ_S_*) is expressed as the sum of two components: *γ_S_^LW^* and *γ_S_^AB^*. The *γ_S_^LW^* component corresponds to SFE associated with long-range interactions such as dispersion, polar, and induction forces, while *γ_S_^AB^* represents the SFE linked to acid-base interactions based on Lewis theory.

(1) $$\gamma_{S}=\gamma_{S}^{LW}+\gamma_{S}^{AB}$$

The Owens–Wendt model (Equation (2)) proposes that the SFE can be divided into two components: *γ_S_^d^*, which accounts for SFE related to dispersion interactions, and *γ_S_^p^*, which encompasses SFE related to polar interactions, including polar, hydrogen bonding, induction, and acid-base interactions.

(2) $$\gamma_{S}=\gamma_{S}^{d}+\gamma_{S}^{p}$$

The gloss of the coatings was evaluated following the procedure outlined in standard ref. [23], using a micro-TRI gloss tester (BYK-Gardner GmbH, Geresried, Germany) at angles of 20°, 60°, and 85°. The reported values represent the average of five measurements, with an experimental error not exceeding 8 degrees.

The Persoz hardness of hybrid coatings on glass plates was measured according to ref. [24] on a pendulum hardness tester (BYK-Gardner GmbH, Germany). The Persoz hardness was defined as the time of oscillations of the pendulum on the material surface to the glass constant (407 s). The values obtained were the mean of five repetitions.

Impact resistance was measured according to ref. [25]. The result is the highest height from which dropping a weight of 1 kg will not damage the coating.

## 3. Results and Discussion

### 3.1. FTIR Spectra of Cationomers

**Figure 3:**
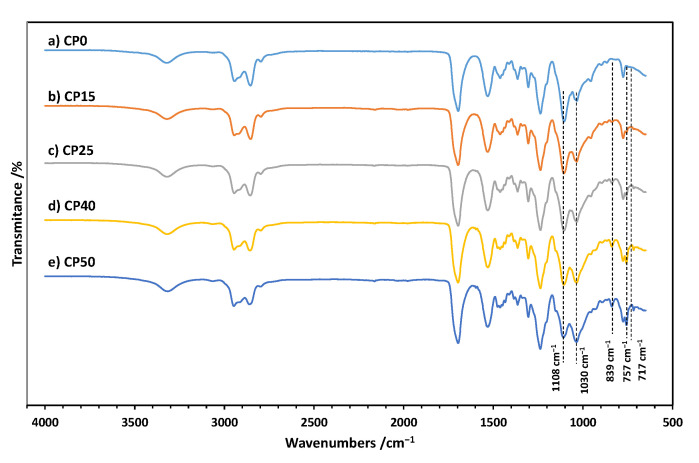
FTIR spectra of CP0 (**a**), CP15 (**b**), CP25 (**c**), CP40 (**d**), and CP50 (**e**).

The structure of the cationomers was confirmed by infrared spectroscopy, and the FTIR spectra for all samples are shown in Figure 3a–e. The complete conversion of the isocyanate groups from IPDI is confirmed by the absence of bands at ca. 2257 cm^−1^. The spectra of all samples show characteristic bands of polyurethanes at ca. 3324 cm^−1^ (-NH stretch), 1695 cm^−1^ (C=O deformation, first amide band), 1526 cm^−1^ (-NH deformation, second amide band), 1245 cm^−1^ (C-N stretching, third amide band), and 765 cm^−1^ (C-N stretching, fourth amide band). Incorporation of TMDS–AGE–DOPA may be evidenced by the appearance of bands at ca. 839 cm^−1^ (CH_3_-Si), 717 cm^−1^, and 757 cm^−1^ (C=C, aromatic) in modified samples. There are also bands in the range of 1580–1620 cm^−1^ related to the C=C bond in the aromatic ring. With an increasing amount of TMDS–AGE–DOPA, the intensity of the absorption band at about 1035 cm^−1^ (Si-O-Si stretching) increases, but decreases at about 1108 cm^−1^ (C-O-C stretching). This is because some of the PTMO chains were replaced by a modifier containing siloxane fragments. There are also other characteristic bands in the range of 2750–3000 cm^−1^ (CH_3_, CH_2_ stretch) in each spectrum.

### 3.2. Differential Scanning Calorimetry (DSC)

**Figure 4:**
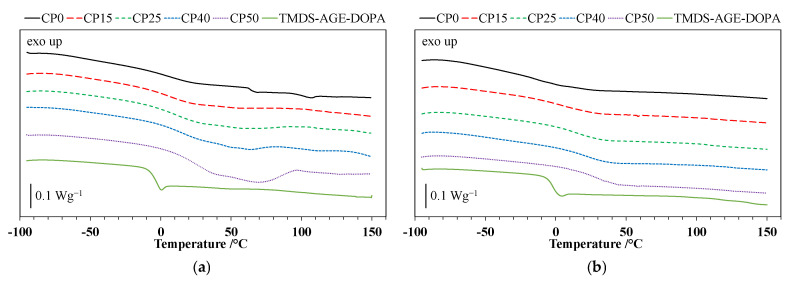
(**a**) DSC curves (first heating) of TMDS–AGE–DOPA and polyurethane cationomers; (**b**) DSC curves (second heating) of TMDS–AGE–DOPA and polyurethane cationomers.

The DSC curves for the polyurethane cationomers and TMDS–AGE–DOPA recorded during the 1st and 2nd heating runs are shown in Figure 4a,b, while the glass transition temperatures (T_g_) are listed in Table 2. Based on the analysis of DSC curves, a single glass transition temperature was observed for the modifier at approximately −5 °C. Nevertheless, the incorporation of TMDS–AGE–DOPA into the polyurethane chain structure increased the T_g_ of the cationomers. The obtained materials exhibit a single-phase, amorphous structure, as evidenced by the presence of a single T_g_ in both runs. The lowest glass transition temperature, measured at −19 °C (2nd heating), corresponds to the unmodified sample, which is related to the transformations in the elastic segments derived from PTMO. However, the glass transition measured from the first run is 1 °C. This temperature shift may be related to the easier possibility of phase separation occurring. The modified samples also exhibit a single glass transition temperature; however, these values shift toward the positive temperature range, between 3 °C and 25 °C. This shift is due to the partial replacement of the long, flexible PTMO chains with TMDS–AGE–DOPA. This modifier has a shorter chain length and contains rigid aromatic rings in its structure, limiting the mobility of the polyurethane cationomer chains. It is worth noting that a small 6% addition of modifier (sample CP15) leads to the greatest increase in the glass transition temperature, reaching 22 °C. Despite its relatively high mass, the modifier is characterized by a short chain. Therefore, a small addition of 6 wt.% effectively replaces 15 mol.% of the more flexible PTMO chain in practice, influencing the glass transition temperature. Comparing the DSC curves after the first and second runs for the modified samples, a slight increase in T_g_ can be seen during the second run. This increase in stiffness may be related to the evaporation of moisture, which has a plasticizing effect.

### 3.3. Thermogravimetric Analysis (TG)

**Figure 5:**
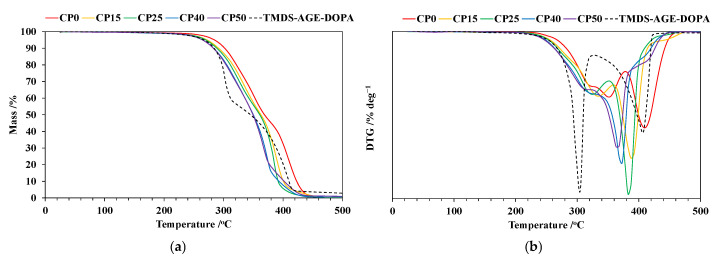
(**a**) TG curves of polyurethane cationomers recorded at 10 °C min^−1^ in nitrogen; (**b**) DTG curves of polyurethane cationomers recorded at 10 °C min^−1^ in nitrogen.

The TG and DTG curves recorded at a heating rate of 10 °C min^−1^ in nitrogen are presented in Figure 5a,b, and Table 2 provides their interpretation. Thermal degradation of TMDS–AGE–DOPA occurs in two main stages in the temperature range of 180–320 °C and 320–520 °C, respectively. The temperature of the maximum mass loss rate at the first stage (T_max1_) is lower compared to all the modified samples. The char yield is very low and does not exceed 3%. Thermal degradation of the unmodified sample is typical for polyurethanes and occurs in two main stages in the temperature range of 200–380 °C and 380–460 °C. The first step of decomposition is associated with the degradation of hard segments derived from IPDI, NMDA, and BD. The temperature of the maximum mass loss rate at this stage (T_max1_) is 351 °C and is accompanied by a mass loss (Δm_1_) of 54.7%. However, at the beginning of that stage up to 330 °C, a plateau is visible, indicating the presence of an additional stage. The second stage is related to the degradation of flexible segments originating from PTMO. The mass loss in this stage (Δm_2_) is 44.8%, and T_max2_ = 409 °C. The thermal degradation of the modified samples follows a slightly different pattern with the addition of modifier, as can be seen from the DTG curves. Only for CP15 and CP25 samples can two separate stages, accompanied by sharp peaks in the DTG curve, be detected. As the modifier content in the sample increases, the stages begin to overlap, and one main thermal degradation stage appears (CP40 and CP50). However, two plateau regions also appear at the initial and final stages of decomposition, suggesting a three-stage thermal degradation process. The introduction of TMDS–AGE–DOPA causes an acceleration of the thermal degradation of cationomers, as confirmed by the reduction of T_max1_ from 351 to 323 °C and T_max2_ from 409 to 382 °C for samples CP0 and CP25, respectively. For the remaining samples, only one temperature can be identified for the main degradation step on the DTG curve, which is even lower and is 370 and 365 °C for samples CP40 and CP50, respectively. Similarly, the temperature of 5% mass loss (T_5%_) is reduced from 283 °C for the unmodified sample to 261 °C for the sample containing 19% modifier. This is because the thermal resistance of the P–C and O=P–O bonds is lower than that of the C–C bond; therefore, the modifier degrades at a relatively lower temperature. In general, the generated phosphoric and polyphosphoric acid could accelerate the dehydration of polyurethanes, thus promoting their charring behavior [10]. However, above a temperature of 400 °C, subsequent reactions related to the degradation of condensed aromatic structures should be expected. The mass residue at 500 °C for the modified samples was in the range of 0.6–1.1%, which may indicate the formation of a charred layer that prevents the fire from spreading, made up of silane moieties. The relatively small amount of degradation residues may be due to a low percentage of phosphorus and silane in the samples. It may also indicate a catalytic effect of the modifier, accelerating the decomposition of the sample toward the production of volatile gases rather than char.

### 3.4. TG-FTIR Analyses

**Figure 6:**
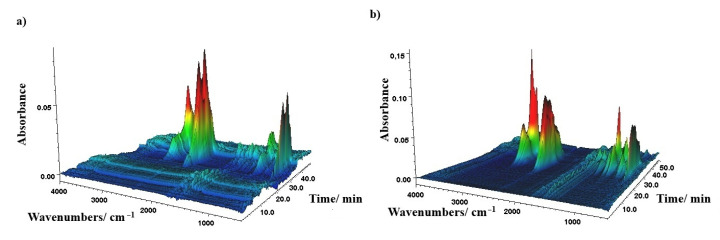
(**a**) 3D FT-IR spectrum of the evolved gases from CP0; (**b**) 3D FT-IR spectrum of the evolved gases from CP50.

**Figure 7:**
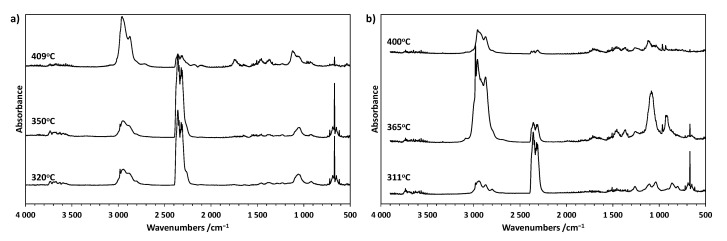
(**a**) FT-IR spectra of gases released from CP0 at several specific temperatures; (**b**) FT-IR spectra of gases released from CP50 at several specific temperatures.

The 3D FTIR spectra for representative CP0 and CP50 samples are presented in Figure 6a,b. Figure 7a,b shows the FTIR spectra of the released gases for several characteristic thermal decomposition temperatures.

The first stage of degradation of the CP0 sample is associated with the release of carbon dioxide as a result of the degradation of rigid polyurethane segments. This is evidenced by the presence of sharp bands at 2358 and 2321 cm^−1^ (stretching mode) and 669 cm^−1^ (bending mode) in the spectrum, arising from vibrations associated with C-O bond corresponding to carbon dioxide. The intensity of these bands is the highest at T_max1_ and then clearly decreases in the second stage. The spectrum also shows vibration bands in the range of 2600–3200 cm^−1^ associated with the stretching vibrations of the C-H bond in -CH_2_ or -CH_3_. The sharp band at 2980 cm^−1^ and broad bands at approx. 1060 and 920 cm^−1^ present in the spectrum may originate from tetrahydrofuran or other ethers or alcohols. Unlike the first stage, in the second stage of the decomposition of the CP0 sample, the presence of carbon monoxide (ca. 2107 and 2180 cm^−1^) and structures containing carbonyl groups (ca. 1749 cm^−1^, >C=O stretching) in the exhaust gases is also observed. Furthermore, the intensity of the bands at approx. 2958 and 1121 cm^−1^ associated with the vibrations of alkyl and ether groups, respectively, increased significantly.

The thermal degradation mechanism of the CP50 sample is more complex. Compared to the thermal degradation of the CP0 sample, the presence of additional vibration bands at about 1260 cm^−1^ related to P=O vibrations can be observed in the exhaust gases. These bands are already visible in the first phase of decomposition, although their intensity is the highest in T_max1_. Additionally, in the main stage of thermal degradation, the highest intensity of the bands is observed at about 2980, 1457, 1363, and 919 cm^−1^, which are characteristic of THF. This indicates the action of TMDS–AGE–DOPA in the gas phase, which accelerates the decomposition of the cationomer sample and the associated shift of T_max2_ toward lower temperatures. As a consequence, the modified samples degrade faster, the stages overlap, and only one extreme is visible on the DTG curve. Ammonia was also identified among the exhaust gases, as evidenced by the characteristic vibration bands at approx. 3333, 966, and 930 cm^−1^. This gas can dilute oxygen and other combustible gases during a fire, lowering its concentration below the levels needed to sustain a fire, as is the case when melamine is used as a filler [26]. Additionally, these gases also absorb heat, cooling the combustion zone. In the final stage of decomposition above 380 °C, degradation of condensed aromatic structures probably occurs, as evidenced by the small amount of residue after degradation. The exhaust gas FTIR spectrum is similar to that of the unmodified sample at T_max2_. However, there are no bands associated with the release of carbon monoxide. Additionally, there are bands originating from the P=O group (1264 cm^−1^), indicating the action of the modifier also in this stage of degradation.

### 3.5. Pyrolysis-Combustion Flow Calorimetry (PCFC)

**Figure 8:**
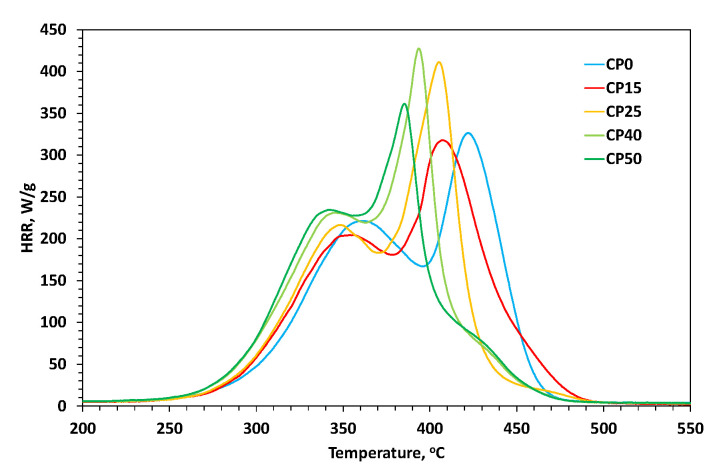
HRR curves of modified cationomers.

Modified and unmodified PU coatings have been subjected to pyrolysis-combustion flow calorimetry (PCFC) testing. This analysis allows one to measure the rate at which the heat of combustion of fuel gases is released by a solid during controlled pyrolysis in an inert gas (N_2_) stream. On the basis of micro combustion calorimetry (MCC) measurements, parameters such as peak of heat release rate (pHRR), temperature of heat release rate (T_PHRR_), heat release capacity (HRC), and total heat release (THR) can be determined. Table 3 presents data from the combustion microcalorimeter, while Figure 8 shows the HRR curves obtained. Based on the collected data, it can be concluded that despite the changing course of HRR curves, the THR for all tested samples is at a very similar level of 28.65 +/− 0.85 (kJ/g)—the differences are no greater than 3% compared to the average. However, the course of the curves itself changes significantly with increasing share of the modifier. For the lowest concentrations (samples CP15 and CP25), the curve is bimodal; for the highest concentrations (CP40 and CP50), it is trimodal; and for the unmodified sample (CP0), the curve becomes bimodal again. It is worth noting that a small additional peak (the third one) that appears for the highest concentrations completely replaces the second peak in the case of unmodified samples. The third additional peak occurring at the highest modifier concentration occurs in the same place where the second of the main heat release peaks occurs for the unmodified material matrix. This is probably related to the fact that at high modifier concentrations during thermal degradation, an ablation layer is formed on the PU surface, inhibiting the material degradation process inside by hindering the transfer of mass and heat from the inside of the sample to the outside and vice versa (the formation of an inorganic layer can be observed from the residue observed in the TG analysis, which increases with the modifier concentration). However, when the outside temperature is further increased, at some point the compounds located under the protective layer are also released and the heat released is recorded. This fact is also confirmed by the correlation in the DTG graph, where the last stage of thermal degradation for the unmodified sample and those for the samples with the highest modifier concentration overlap, during which a mass loss is observed until it reaches a constant, final value. For samples with lower modifier concentrations, the degradation process ends at lower temperature values, which may indicate that the ablation layer formed for them is too thin and therefore ineffective in blocking the transfer of mass and energy outside the sample.

For all samples, the first peak is in a similar range of HRR values (205–235 W/g) and occurs at a similar temperature value (range of 343–361 °C). The discussed peak shifts most significantly toward lower temperatures for the CP50 sample, while assuming the highest value of the HRR parameter. A similar relationship can be observed for the second peak, which shifts most notably toward lower temperatures for the CP50 sample—the highest modifier concentration contributes to the greatest change. As already mentioned above, the second peak, with increasing modifier concentration, begins to shift toward lower temperature values (from sample CP15 to CP50), and for the unmodified sample (CP0), it occurs well above the temperature assigned for sample CP15—it occurs near the place where a small, third, additional peak appears for samples CP40 and CP50. Therefore, it can be assumed that with increasing modifier concentration (CP40), an additional thermal degradation process begins to occur (the appearance of a new peak in the graph). This process clearly stands out for the unmodified sample with the highest HRR values in the graph. Hence, the presence of the modifier in the PU matrix probably inhibits this additional thermal degradation process, which dominates for unmodified samples. Samples with the modifier in the PU matrix show typical behavior for flame retardant compounds, shifting the pHRR value toward lower temperature values [19,27]. In turn, the lack of a modifier in the structure causes a significant change in the course of the HRR curves. A similar observation can be made based on the HRC values, which start to increase with increasing modifier concentration and decrease slightly again for the two unmodified samples.

### 3.6. Contact Angle and Surface Free Energy

The contact angle (CA) and surface free energy parameters (SFE) of the crosslinked coatings are summarized in Table 4. The water contact angles for all samples are below 90 degrees, confirming the hydrophilic nature of the cationomer coatings. The unmodified sample CP0 exhibits the highest contact angles for all liquids tested. However, as the modifier content increases, the contact angles decrease, indicating enhanced hydrophilicity. This change is attributed to the higher share of polar P=O bonds introduced by TMDS–AGE–DOPA. In our previous studies on cross-linked cationomers, it was observed that the addition of a similar modifier resulted in an increase in hydrophobicity [19]. Notably, the hydrophobic effect of siloxane structures is not dominant in this type of cationomers. Additionally, a trend was observed where the contact angles increased with the rising polarity of the measuring liquids used for testing each coating.

Two methods were used to calculate the surface free energy, i.e., van Oss–Good (vOG), and also to verify the results using the Owens–Wendt (OW) method. The SFE values for the cationomer samples are relatively high, ranging from 37.6 to 44.0 mJ/m^2^. As the modifier content increases, the surface free energy increases, with the most hydrophilic sample being CP50, which has an SFE of 44.0 mJ/m^2^. Using the vOG method, the total SFE value is predominantly influenced by the *γ_S_^LW^* component, representing long-range interactions. In contrast, the OW method indicates that the total surface free energy is primarily affected by the polar component *γ_S_^p^*. The share of this component exceeds 97% in the case of SFE calculations using the diiodomethane aqueous liquid system, which shows a significant difference in polarity. Furthermore, the *γ_S_^p^* component increases with increasing amount of TMDS–AGE–DOPA, which further confirms the hydrophilic nature of the samples. The results from both the van Oss–Good and Owens–Wendt methods show a good correlation, confirming the reliability of the calculations. The obtained coatings can be used on hydrophilic surfaces such as textiles, glass, or wood. However, the acidic nature of cationomers limits their application on surfaces made of ferrous metals. Additionally, their relatively high surface free energy and lack of chemical cross-links can make them prone to water damage.

### 3.7. Performance Properties of the Polyurethane Cationomer Coatings

The gloss of the cationomer coatings at 20, 60, and 85°, as well as the Persoz hardness and impact resistance, are presented in Table 5.

The samples are characterized by high gloss, above 100 GU, measured for each geometry of 20°, 60°, and 85°. Adding a small amount of modifier (6%) increases gloss, but further increasing the amount of modifier reduces gloss. This may be due to the increased content of siloxane segments, which cause a reduction in gloss.

The coatings are relatively soft, with a Persoz hardness ranging from 0.05 to 0.32. As the modifier content increases, the hardness also increases. This is attributed to the aromatic structure and shorter chain length of TMDS–AGE–DOPA, which contribute to stiffening the cationomer structure. These findings are consistent with the DSC results, where the introduction of the modifier resulted in an increase in the glass transition temperature. Nevertheless, the hardness of the obtained coatings is lower compared to the flame-retardant poly(urethane-acrylic) cross-linked cationomers [19].

The impact strength of the samples depends on the amount of TMDS–AGE–DOPA. The unmodified sample and the sample containing 6 wt.% of modifier (CP15) demonstrated excellent impact strength (100 cm). Further increasing the amount of modifier caused a decrease in impact strength by up to 75% in the case of samples containing more than 15% of modifier (CP40 and CP50). As with hardness, this is related to the presence of aromatic segments, which also influence brittleness.

## 4. Conclusions

Polyurethane cationomers modified with TMDS–AGE–DOPA were successfully obtained. The polymers exhibit an amorphous single-phase structure, with the unmodified sample showing the lowest glass transition temperature (−19 °C) due to the presence of flexible segments derived from PTMO. Modification with TMDS–AGE–DOPA increases T_g_ (up to 25 °C) by introducing shorter chains and rigid aromatic rings, significantly reducing chain mobility. Modification with TMDS–AGE–DOPA also reduces the thermal stability of the samples, as indicated by a reduction in the 5% mass loss temperature from 283 to 264 °C. This effect results from the action of TMDS–AGE–DOPA in the gas phase and is associated with the release of gases such as THF, ammonia, and CO_2_. The addition of the TMDS–AGE–DOPA to the polyurethane coatings significantly influences their combustion behavior by shifting the peak heat release rate (pHRR) and temperature of heat release rate (T_PHRR_) to lower temperatures, which is typical for flame-retardant materials. Although the total heat release (THR) remains similar across all samples, the modifier inhibits additional thermal degradation processes, reducing peak HRR values compared to the unmodified samples and improving the flame resistance of the material. The addition of TMDS–AGE–DOPA decreases the water contact angle from 89 to 85°, increasing the hydrophilicity of the cationomer coatings. Furthermore, the surface free energy of the samples rises with increasing modifier content, from 37.9 mJ/m^2^ to a maximum of 44.0 mJ/m^2^ for the most hydrophilic sample, CP50. The addition of a small amount of modifier (6%) increases the gloss of the coatings, while a higher modifier content reduces gloss, which is in the range of 144–166 GU at 60°. The modifier also increases the relative hardness of the coatings from 0.05 to 0.32; however, excessive amounts (more than 15%) reduce impact strength by 75% due to the increased brittleness caused by the aromatic segments. These materials are promising for flame-retardant coatings for, e.g., textiles, glass, or wood. However, a limitation is their brittleness at high modifier content. Future research should focus on developing cationomers with optimized structures that combine high flame retardancy with suitable mechanical properties.

## Figures and Tables

**Table 1:** Chemical composition of polyurethane cationomers modified with TMDS–AGE–DOPA.

Sample	CP0	CP15	CP25	CP40	CP50
IPDI/mol	4	4	4	4	4
PTMO/mol	1	0.85	0.75	0.60	0.50
TMDS–AGE–DOPA/mol	0	0.15	0.25	0.40	0.50
NMDA/mol	1	1	1	1	1
HCOOH/mol	1	1	1	1	1
BD/mol	2	2	2	2	2
IPDI/wt.%	40%	40%	41%	41%	41%
PTMO/wt.%	45%	38%	34%	28%	23%
TMDS–AGE–DOPA/wt.%	0%	6%	9%	15%	19%
NMDA/wt.%	5%	5%	5%	6%	6%
HCOOH/wt.%	2%	2%	2%	2%	2%
BD/wt.%	8%	8%	8%	8%	8%

**Table 2:** Interpretation of DSC, TG, and DTG curves of polyurethane cationomers.

Sample	TMDS–AGE–DOPA	CP0	CP15	CP25	CP40	CP50
T_g_/°C (1st heating)	−5	1	2	8	13	22
T_g_/°C (2nd heating)	−3	−19	3	12	17	25
T_onset_/°C	271	288	276	272	268	264
T_5%_/°C	265	283	270	268	262	261
T_max1_/°C	301	351	333	323	-	-
Δm_1_/%	44.5	24.3 *	47.7	44.9	36.1 *	31.1 *
		30.4				
T_max2_/°C	407	409	388	382	370	365
Δm_2_/%	53.0	44.8	48.7 3.0 *	53.6 1.0 *	52.2 11.1 *	53.2 14.6 *
Remaining mass/%	2.5	0.5	0.6	0.5	0.6	1.1

* plateau in DTG curve.

**Table 3:** Results of PCFC analysis.

Sample	Peak I pHRR [W/g]	Peak I T_PHRR_ [°C]	Peak II pHRR [W/g]	Peak II T_PHRR_ [°C]	Peak III pHRR [W/g]	Peak III T_PHRR_ [°C]	ηc [J/g·K]	THR [kJ/g]
CP0	221	361	327	422	-	-	474	29.3
CP15	205	356	318	408	-	-	382	29.5
CP25	217	348	411	405	-	-	500	28.6
CP40	231	346	427	394	74	428	475	28.7
CP50	235	343	361	385	78	430	530	27.8

**Table 4:** The CA and SFE parameters of polyurethane cationomers.

Sample	CP0	CP15	CP25	CP40	CP50
Diiodomethane CA/deg	43	41	40	35	30
Formamide CA/deg	79	70	69	58	59
Water CA/deg	89	87	87	86	85
SFE parameters—vOG method					
*γ_S_^LW^*/mJ m^−2^	37.9	37.2	37.7	41.8	44.0
*γ_S_*^+^/mJ m^−2^	0.0	0.0	0.0	0.1	0.0
*γ_S_*^−^/mJ m^−2^	11.0	2.6	3.3	2.1	3.0
*γ_S_^AB^*/mJ m^−2^	0.0	0.0	0.0	0.8	0.0
*γ_S_*/mJ m^−2^	37.9	37.2	37.7	42.6	44.0
SFE parameters—OW method Water-Diiodomethane liquids					
*γ_S_^d^*/mJ m^−2^	1.2	0.7	1.0	0.9	0.9
*γ_S_^p^*/mJ m^−2^	36.4	38.0	38.0	40.6	43.1
*γ_S_*_/_mJ m^−2^	37.6	38.7	39.0	41.5	43.9
SFE parameters—OW method Formamide-Diiodomethane liquids					
*γ_S_^d^*/mJ m^−2^	0.0	1.5	1.7	4.8	3.4
*γ_S_^p^*/mJ m^−2^	39.0	36.8	37.2	36.9	40.3
*γ_S_*_/_mJ m^−2^	39.0	38.3	38.9	41.7	43.7

**Table 5:** Performance properties of cationomer coatings.

Sample	CP0	CP15	CP25	CP40	CP50
Gloss at 20°/GU	138	173	161	153	136
Gloss at 60°/GU	146	166	159	154	144
Gloss at 85°/GU	101	110	104	107	102
Persoz hardness/-	0.05	0.07	0.10	0.19	0.32
Impact resistance/cm	100	100	50	25	25

## Data Availability

Data are contained within the article.
